# Molecular Characterization and Expression Pattern of Tripartite Motif Protein 39 in *Gallus gallus* with a Complete PRY/SPRY Domain

**DOI:** 10.3390/ijms12063797

**Published:** 2011-06-09

**Authors:** Chunqing Pan, Heng Zhao, Lin Shen, Jiping Sheng

**Affiliations:** 1 College of Food Sciences, China Agricultural University, Beijing 100080, China; E-Mails: pcq201005@yahoo.com.cn (C.P.); shen5000@cau.edu.cn (L.S.); 2 Key Laboratory for Feed Biotechnology of the Ministry of Agriculture, Feed Research Institute, Chinese Academy of Agricultural Sciences, Beijing 100081, China

**Keywords:** chicken, tripartite motif protein 39, B30.2 domain

## Abstract

Members of tripartite motif (TRIM) proteins in mammals play important roles in multiple cellular processes in the immune system. In the present study we have obtained the chicken TRIM39 with the insertion of a base A at position 1006 bp, compared to the sequence in the NCBI database (Accession No: NM 001006196), which made TRIM39 fulfill the TRIM rule of domain composition with both PRY, and SPRY domains. The open reading frame consisted of 1392 bp encoding 463 amino acid residues. The amino acid sequences of TRIM39 protein in mammals were highly similar (from 91.48% to 99.61%), while chicken TRIM39 had relatively low homology with mammals (from 29.2% to 39.59%). Real time RT-PCR indicated that the mRNA expression level of TRIM39 was the highest in spleen, with a lower expression in liver, brain, and lung, suggesting it might be an important protein participating in the immune system.

## 1. Introduction

Tripartite motif (TRIM) proteins are also known as RBCC proteins as they comprise a RING domain, one or two B-boxes and a predicted coiled-coil region from the N- to C-terminus [1]. The RING domain is a specialized zinc finger consisting of 40–60 residues that binds two zinc atoms. Studies have demonstrated that the RING domain of many TRIM proteins have ubiquitin E3 ligase activity [2,3]. B-boxes bind zinc atoms similar to the RING domain, and are found exclusively in TRIM proteins, although no function has been clearly assigned to these domains. The coiled-coil domain mainly participates in the homo-oligomeric and hetero-oligomeric interaction to form large protein complexes. Tripartite motif protein was first found in *Xenopus laevis* as a novel zinc finger nuclear phosphoprotein (xnf7) [4]. At present, tripartite motif proteins and their homologues have been identified in many species from primates to eels [5], teleost fishes [6] and nematodes [7]. To date, many genes and pseudogene encoded tripartite motif proteins have been found in human and this is ever increasing [8].

The tripartite motif proteins play multiple roles in a series of important cellular processes, including cell proliferation, differentiation; development, oncogenesis, apoptosis, and antiviral defense [8–10]. Many pathological symptoms, from Mendelian genetic diseases to cancer development and viral infection, frequently have a relationship with tripartite motif proteins [11–13]. In addition, TRIM family members represent a fast-growing list of antiviral molecules involved in the innate immunity. However, TRIM proteins need to be further studied to elucidate all the functions of this gene family [8,14]. In chicken, a cluster of TRIM-B30.2 genes including TRIM39 in the chicken MHC B locus has been identified flanking the chicken *BF/BL* region and may be a candidate for genes affecting infectious disease [15,16].

In order to gain a better understanding of the chicken TRIM39, we have obtained the full-length cDNA sequence of TRIM39 and found an insertion of a base A at the position of 1006 bp compared to the sequence of TRIM39 in the NCBI database (NM-001006196) [15]. Annotation of genetically mobile domains and the analysis of domain architectures found that the TRIM 39 proteins fulfill the TRIM rule of domain composition and has both PRY and SPRY domains. In addition, real time RT-PCR indicated that the amount of tripartite motif protein 39 is different in various tissues. The mRNA expression level was the highest in spleen and with a lower expression in liver, brain, and lung, suggesting that TRIM39 protein might be an interesting candidate affecting infectious disease.

## 2. Results and Discussion

### 2.1. Defining the Complete TRIM 39 Gene in Gallus Gallus

**Figure 1:**
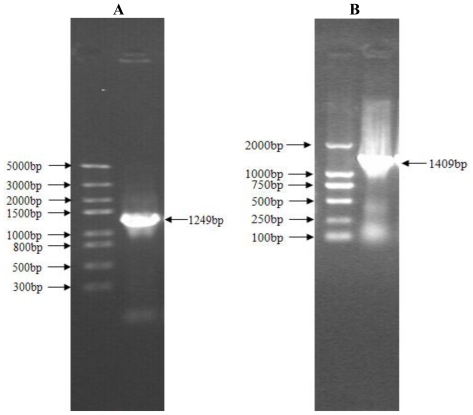
Nested PCR amplification of TRIM39. **(A)** PCR product amplified with primer 1, primer 2 as outer nested PCR primers and primer 3, primer 4 as inner nested PCR primers; **(B)** PCR product amplified with primer 1, primer 5 as outer nested PCR primers and primer 3, primer 6 as inner nested PCR primers. In each picture, the left lane shows DNA marker and the right lane shows PCR product.

In order to clone cDNA from the TRIM 39 gene, we performed a nested PCR by using primer 1 and 2 as outer PCR primers, primer 3 and 4 as inner PCR primers (Table 1). A PCR product of 1249 bp was obtained (Figure 1A) with an insert of a base A in position 1006 bp compared to the TRIM39 sequence of NCBI (NM 001006196) after DNA sequencing. Due to this inserted base A, the open reading frame (ORF) of TRIM39 has been shifted. Subsequently, we designed new reverse primers (primer 5 and 6, Table 1), a PCR product of 1409 bp was obtained by using primer 1, primer 5 as outer PCR primers, primer 3 and primer 6 as inner PCR primers (Figure 1B).

### 2.2. Domain Composition of Chicken TRIM 39 Protein

**Figure 2:**
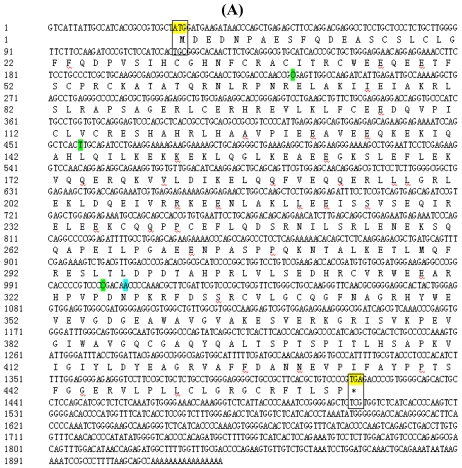

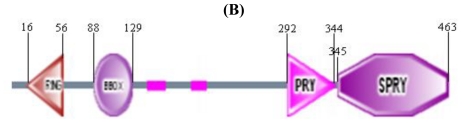
**(A)** cDNA sequence and the predicted amino acid sequence of TRIM39 in chicken, *Gallus gallus*. The boxes represent the initial codon and the stop codon. Green shaded areas represent bases different from the sequence of TRIM39 from NCBI. Blue shaded areas represent the insert base A compared to the sequence of TRIM39 from NCBI; **(B)** Schematic representation of domain architectures of the new TRIM39 predicted protein sequence by SMART online service.

The full-length cDNA sequence of TRIM 39 was analyzed by using DNAMAN 6.0 software. The ORF contained 1386 bp and encoded for a deduced protein of 463 amino acids (Figure 2A). The predicted protein sequence was used to request the SMART service to show the annotation of genetically mobile domains and the analysis of domain architectures (Figure 2B). We found that the new TRIM39 predicted protein encoded a SPRY domain which does not appear in the TRIM39 protein deduced from NCBI. To search for TRIM 39 proteins in other species, we screened NCBI and obtained the NCBI accession number and domain architectures in human, mouse, rat, pig, *etc.* The amino acid sequences of TRIM39 protein in mammals were highly similar with each other (from 91.48% to 99.61%), while the chicken TRIM39 had lower homology with mammals (from 29.2% to 39.59%).

### 2.3. Alignment of Chicken TRIM 39 with Other Species

**Figure 3:**
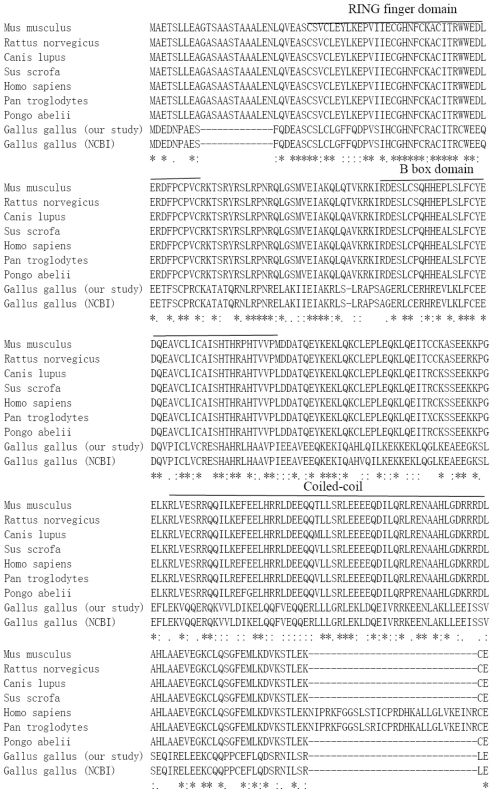

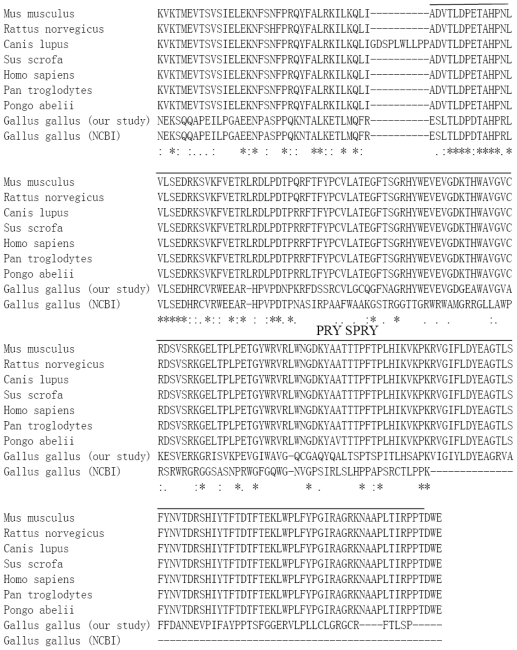
Sequence alignment of the predicted amino acid of TRIM39 from different species. Identities are denoted by an asterisk (*), conserved similarities by dots (:) and (.), respectively, and deletions are introduced for optimal alignment by a dash (–).

The chicken TRIM39 protein sequences were analyzed by ClustalW, the RING domain of chicken TRIM 39 has the C-X2-C-X(9-39)-C-X(1-3)-H-X(2-3)-C-X2-C-X(4-48)-C-X2-C structure (at position 29–69), very similar to other species, including human and mouse (Figure 3). Though chicken TRIM39 has a low similarity with mammals, the TRIM39 predicted in our study has a higher similarity compared to the TRIM39 retrieved from ENSEMBL [16] (Table 2). The majority of the TRIM 39 proteins fulfill the TRIM rule of domain order and composition, RING, B-box, CC, C-terminal domains which have both PRY and SPRY domains. The domain architecture of chicken TRIM 39 deduced from our sequence data has both PRY and SPRY domains, while that from NCBI contains only PRY domain (Table 3).

### 2.4. Tissue Expression Analysis

**Figure 4:**
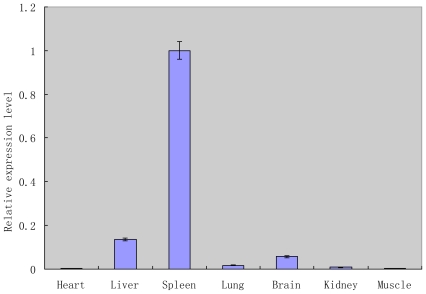
TRIM39 mRNA expression in various tissues of chicken. TRIM39 mRNA expression levels were measured by real-time PCR as described in materials and methods. Data show the means of three independent experiments. Error bars denote S.D. of three independent experiments.

Real-time PCR was performed to detect the mRNA expression levels of the TRIM39 gene in multiple tissue of chicken. Our results showed that the relative expression levels of TRIM39 mRNA are quite different among different tissues. The highest level of TRIM39 mRNA was detected in spleen, while liver, brain had a reduced amount, and lung, kidney, muscle, and heart had the lowest level (Figure 4).

### 2.5. Discussion

In the present study, we obtained the full-length cDNA sequence of TRIM39, and sequence alignment showed an insertion of a base A at the position of 1006 bp by comparing with the sequence of TRIM39 in the NCBI database [15]. We have applied high fidelity DNA polymerase for PCR amplification to avoid the error caused by PCR. The inserted base A made the translated protein of TRIM 39 obtained in our study encoded RING, B-box, and PRY/SPRY (B30.2). It seems that chicken TRIM 39 protein deduced from NCBI is “incomplete” without SPRY domain. The missing A occurred in the middle of an exon at position 1006 (Figure 2A), and is not a part of a run of As, suggesting it might not be difficult to sequence; this could be due to the NCBI sequence being derived from Red Jungle Fowl and our sequence data was from indigenous chickens.

Further analysis showed that the TRIM39 predicted in our study had a higher similarity (99.35%) to the TRIM39 retrieved from other published data (ENSGALP00000000151) [16]. Only three amino acid differences were found at position of 143, 248, 336, respectively. Comparison of the TRIM39 protein sequences from different species reveals relatively high sequence homology. The amino acid sequences of TRIM39 protein in mammals are highly similar with each other, from 91.48% to 99.61%, while the chicken TRIM39 had lower homology with mammals (from 29.2% to 39.59%). The analysis of domain architectures shows that the new TRIM39 predicted protein encoded a SPRY domain which does not appear in the TRIM39 protein deduced from NCBI. The B30.2 domain was first identified in the human class I major histocompatibility complex region while SPRY domain was first identified in a *Dictyostelium discoidueum* kinase splA and mammalian Ca^2+^ release channels ryanodine receptors [17,18]. These two domains are highly similar in sequence, although the SPRY domains are shorter at the N-terminus than the B30.2 domains. In many cases, a PRY domain often exists right next to the N-terminus of the SPRY domain which appears as a sub-domain of the B30.2 domain [19]. There are many proteins that contain SPRY or B30.2 domain which participate in a wide range of different biological processes [20,21]. Studies have demonstrated that the crystal structure of TRIM21 PRY/SPRY in complex with its target IgG Fc reveals a canonical binding interface comprised of two discrete pockets formed by antibody-like variable loops [22].

It is interesting that the RING domains all feature the C-X2-C-X(9-39)-C-X(1-3)-H-X(2-3)-C-X2- C-X(4-48)-C-X2-C structure. The RING domains in several TRIM family members have demonstrated ubiquitin E3 ligase activity. In human, it has been found that TRIM39 could significantly extend the half-life of MOAP-1 stability by inhibiting its polyubiquitination process. It could be that the RING domain of chicken TRIM39 also has ubiquitin E3 ligase activity and plays an important role during TRIM39 protein functioning. We also investigated the mRNA expression levels of the TRIM39 gene in multiple tissues of chicken. Our results showed that the relative expression levels of TRIM39 mRNA are quite different among different tissues. The TRIM39 mRNA is highly expressed in spleen, with less expression in liver, brain, and lung, insinuating that the chicken TRIM39 might be an important protein participating in the immune system.

## 3. Experimental Section

### 3.1. Experimental Animal and Reagents

Three yellow broilers were purchased from a local market in Beijing. The animals were sacrificed, and various tissues including liver, brain, kidney, lung, heart, spleen, pancreas and muscle were dissected and immediately frozen with liquid nitrogen after Phosphate buffered saline (PBS) washes to remove blood. The tissue samples were then stored in an −80 °C freezer or immediately used for extraction of RNA as described below. The PCR TeqMix, DNA Marker and pEASY-T1 Simple Cloning Kit were purchased from TransGen Biotechnology Inc. The DNA gel extraction kit was obtained from the New Industry Biotechnology Inc. The oligo primer synthesis was provided by Beijing AuGCT biotechnology Co., Ltd. The DNA sequencing services was provided by The Beijing Genomics Institute.

### 3.2. Total RNA Isolation

RNA isolation was performed from 30 mg of fresh tissues using the SV Total RNA Isolation System from Promega. Purification steps were processed according to the technical manual of SV total RNA isolation. The RNA quality was checked on an agarose gel and the yield was obtained by spectrophotometry.

### 3.3. Reverse Transcription Polymerase Chain Reaction

The RT-PCR reaction was performed in two steps (TaKaRa Biotechnology Dalian Co., Ltd, Dalian, China). First, approximately 1 μg of total RNA and 2.5 μM of Oligo dT_18_ were added in a micro-centrifuge tube, then DEPC-treated water was added to a final volume of 10 μL. After brief centrifugation, the mixture was heated to 70 °C for 10 min and chilled on ice for at least 1 min. Second, 5 × M-MLV buffer, 0.2 μM dNTPs, 20 U of RNase inhibitor, and 50 U M-MLV reverse transcriptase were added in the mixture, and the RT conditions were carried for 15 min at 42 °C, 60 min at 70 °C and 5 min at 4 °C.

According to the TRIM39 gene cDNA sequence information of *Gallus gallus* (Accession NO: NM_001006196) from the NCBI database, we designed the primers with Primer Premier 5.0 software. The coding sequence of TRIM39 was amplified by nested PCR using the *Gallus gallus* spleen cDNA as templates. All the experimental primers used in this study are listed in Table 1.

### 3.4. Real-Time RT-PCR Assay

Real-time RT-PCR reactions were performed using the SYBR green reagent from TOYOBO (TOYOBO Co., Ltd, Osaka, Japan) and Chromo 4 Real-Time PCR Detector (BIO-RAD, California, CA, USA), according to the manufacturer’s instructions. Oligonucleotides used for real-time RT-PCR are indicated in Table 1. All reactions were performed in triplicate. Data analysis was performed as described in the ABI PRISM 7700 sequence detection bulletin #2 from Applied Biosystems, following the 2^−Δ Δ Ct^ method. Amplification of GAPDH was used as internal reference.

### 3.5. Prediction of Protein Structure and Analysis of Sequence Homology

The full-length cDNA sequence and the ORF were determined using DNAMAN 6.0 software. The publicly available Expert Protein Analysis System (ExPASy) [23] was used to analyze the characteristics of the predicted protein sequence. The protein structure was predicted by SMART online software [24]. Sequences alignments were determined by bl2seq in NCBI, and the multi-sequence comparison was conducted using ClustalW software.

## 4. Conclusions

Our study showed that chicken TRIM39 has the insertion of a base A at position 1006 bp, compared to the sequence of TRIM39 in the NCBI database, and this inserted base A made the translated protein of our obtained sequence encoding the complete motif of RING, B-box, PRY, and SPRY. Though chicken TRIM39 had a low similarity with mammals, the TRIM39 predicted in our study had a higher similarity compared to the TRIM39 retrieved from ENSEMBL. Further studies indicated that the amount of tripartite motif protein 39 differs in various tissues. The mRNA expression level was the highest in spleen, with a lower level expressed in liver, brain, and lung.

## Figures and Tables

**Table 1:** Experimental primers a).

Name	Primer sequence (5′→3′)
Primer 1	CCGTCGCTATGGATGAAG
Primer 2	GTGGGAGGGTACGCAAAA
Primer 3	CGCGGATCCATGGATGAAGATAACCCAGCTG
Primer 4	ACGCGTCGACCTTTGGGGGCAGAGTGCAG
Primer 5	TGTCCAAACTGTCGCCTCT
Primer 6	ACGCGTCGACGCGGGGACAGCGTGAAGCGG
Primer 7	CTCCTGCTCCCTCTGCTTG
Primer 8	GGTTTCCTCCTGTTCCTCCC
Primer 9	ATGGCATCCAAGGAGTGAGC
Primer 10	CAGAACTGAGCGGTGGTGAA

a) The underline represents the restricted enzyme cutting site of *BamH I* and *Sal I*. The protective bases were chosen according to the principle of primer design. Primer 7, 8, 9, 10 are primers for TRIM39 and GAPDH used in the real-time RT-PCR assay, respectively.

**Table 2:** The comparison of TRIM39 proteins from different species.

	Dog	Human	House mouse	Chimpanzee	Sumatran orangutan	Norway rat	Pig	Chicken (NCBI)	Chicken (Ensembl)
Human	91.67%								
House mouse	95.38%	91.89%							
Chimpanzee	91.48%	99.61%	91.89%						
Sumatran orangutan	96.39%	93.44%	96.72%	93.24%					
Norway rat	95.38%	91.89%	99.18%	91.89%	96.72%				
Pig	97.39%	94.02%	97.75%	93.82%	98.98%	97.75%			
Chicken (NCBI)	30.28%	29.20%	30.63%	29.20%	30.97%	30.63%	30.97%		
Chicken (Ensembl)	38.40%	36.92%	39.18%	36.92%	38.78%	39.18%	39.18%	73%	
Chicken (our study)	38.80%	37.31%	39.39%	37.12%	39.18%	39.59%	39.59%	73%	99.35%

**Table 3:** The NCBI accession number and domain architectures of TRIM39 from different species.

Species	NCBI Accession No.	The domain architectures of TRIM39
Canis lupus	XP_855659	RING, BBOX, BBC, PRY, SPRY
Homo sapiens	NP_067076	RING, BBOX, BBC, PRY, SPRY
Mus musculus	NP_077788	RING, BBOX, PRY, SPRY
Pan troglodytes	NP_001065263	RING, BBOX, BBC, PRY, SPRY
Pongo abelii	NP_001125160	RING, BBOX, PRY, SPRY
Rattus norvegicus	NP_998727	RING, BBOX, BBC, PRY, SPRY
Sus scrofa	NP_001121951	RING, BBOX, BBC, PRY, SPRY
Gallus gallus (NCBI)	NP_001006196	RING, BBOX, PRY
Gallus gallus (Ensembl)	ENSGALP00000000151	RING, BBOX, PRY, SPRY
Gallus gallus (our study)		RING, BBOX, PRY, SPRY
